# Auricular magnetic bead acupressure improves postpartum sleep quality and fatigue, and reduces epidural-related maternal fever in primiparas: a randomized controlled trial

**DOI:** 10.3389/fmed.2026.1788646

**Published:** 2026-03-13

**Authors:** Jian Tang, Qian Zhai, Yutong Liang, Zhenhe Yu, Jing Song, Zhujing Zhang, Yaqi Wang, Ying Chen, Zhonglu Jian

**Affiliations:** 1 Department of Anesthesiology, The First Affiliated Hospital of Guizhou University of Traditional Chinese Medicine, Guiyang, Guizhou, China; 2 School of Nursing, Guizhou University of Traditional Chinese Medicine, Guiyang, Guizhou, China; 3 Department of Anesthesiology, The Second Affiliated Hospital of Guizhou University of Traditional Chinese Medicine, Guiyang, Guizhou, China; 4 School of Anesthesia, Guizhou Medical University, Guiyang, Guizhou, China

**Keywords:** auricular magnetic bead acupressure, epidural-related maternal fever, postpartum fatigue, postpartum sleep disturbance, primiparas

## Abstract

**Purpose:**

Postpartum sleep disturbances and severe fatigue are prevalent health concerns among primiparas and may be associated with neuroendocrine and immune activation during the perinatal period. Auricular magnetic bead acupressure (AMBA), a non-invasive therapy rooted in traditional Chinese medicine, has been reported to provide analgesic and anti-inflammatory effects and to modulate autonomic function, and may offer an adjunctive approach to improve postpartum sleep disturbances and fatigue.

**Patients and methods:**

This prospective randomized controlled trial included 144 primiparas who underwent epidural analgesia. Participants were randomly allocated to either the AMBA intervention group (AMBA combined with Patient-Controlled Epidural Analgesia, PCEA) or the control group (PCEA). The primary outcomes assessed were postpartum sleep quality, evaluated using the Athens Insomnia Scale (AIS), and fatigue severity, assessed via the Postpartum Fatigue Scale (PFS). Secondary outcomes encompassed the incidence of epidural-related maternal fever (ERMF), levels of inflammatory cytokines (IL-6, IL-1β), and sleep-related biomarkers such as melatonin and serotonin (5-HT).

**Results:**

A total of 122 parturients completed the study. The AMBA group demonstrated a significantly lower AIS score on postpartum day 5 compared with the control group (4.72 ± 2.77 vs. 6.03 ± 3.13, *p* = 0.0064), as well as a significantly enhanced fatigue score (16.13 ± 2.91 vs. 20.40 ± 0.89, *p* = 0.0003). Additionally, the AMBA group exhibited higher postpartum levels of melatonin and 5-HT, lower levels of IL-6 and IL-1β, reduced incidence of ERMF (8.19% vs. 24.59%, *p* = 0.0265), a shorter second stage of labor, and lower total analgesic consumption and oxytocin dose (all *p* < 0.05).

**Conclusion:**

Auricular magnetic bead acupressure effectively enhances postpartum sleep quality and alleviates fatigue in primiparas receiving epidural analgesia, while also reducing the incidence of ERMF. The underlying mechanism may involve the modulation of neuroendocrine and immune system functions. AMBA constitutes a safe, non-invasive, and non-pharmacological intervention strategy that is well-suited for comprehensive perinatal management.

**Clinical trial registration:**

http://itmctr.ccebtcm.org.cn/, identifier ITMCTR2025002105.

## 1 Introduction

Postpartum sleep disturbances and severe fatigue are prevalent health concerns among primiparous women, significantly compromising intrapartum safety, mother-infant interactions, and long-term quality of life. These conditions, often co-occurring with labor pain, negatively impact maternal and neonatal outcomes (1, 2). The intense pain, physiological stress, and associated medical interventions, such as epidural analgesia, during labor can substantially activate the hypothalamic–pituitary–adrenal (HPA) axis and systemic pro-inflammatory cytokine networks (3, 4). This activation of the neuroendocrine and immune systems during the perinatal period represents a critical pathophysiological basis for postpartum sleep disorders and fatigue (5, 6).

Notably, this pro-inflammatory state is also central to the mechanism of epidural-related maternal fever (ERMF) (7). ERMF is a common obstetric complication, with an incidence of 15–30% among parturients receiving analgesia. It not only increases the likelihood of interventions such as instrumental delivery but also potentially affect neonatal outcomes. Therefore, inflammation can be viewed as a common pathological nexus connecting perinatal pain, fever, and sleep-fatigue disturbances (8, 9). Despite this understanding, current clinical practices for parturients undergoing epidural analgesia primarily emphasize symptomatic relief of pain and fever. There is a notable absence of early intervention strategies that are both safe and effective in modulating inflammation to concurrently enhance postpartum sleep and reduce fatigue.

Auricular Magnetic Bead Acupressure (AMBA), a non-invasive therapy rooted in traditional Chinese medicine meridian theory, aims to regulate bodily functions through the stimulation of specific auricular points (10, 11). Research suggests that AMBA exerts multiple beneficial effects, including analgesic, anti-inflammatory, and autonomic nerve regulation properties (12). This evidence provides a theoretical basis for its potential application in addressing perinatal neuro-immune-endocrine imbalances, thereby offering a promising approach to simultaneously improve postpartum sleep and fatigue while preventing intrapartum fever. Based on this premise, we propose that AMBA, due to its anti-inflammatory and neuromodulatory properties, may serve as an effective non-pharmacological intervention to enhance postpartum sleep quality and reduce fatigue in parturients undergoing epidural analgesia. Additionally, its anti-inflammatory effects may indirectly decrease the incidence of epidural-related maternal fever (ERMF).

Consequently, the primary objective of this randomized controlled trial is to assess the efficacy of AMBA in improving postpartum sleep quality and reducing fatigue severity among primiparas. The study will also investigate its potential preventive impact on ERMF. Furthermore, we will preliminarily explore the mechanisms of AMBA by systematically monitoring dynamic changes in body temperature, inflammatory cytokines (such as IL-6 and IL-1β), and key sleep-related biomarkers (including melatonin and serotonin). The anticipated findings are expected to contribute novel evidence and insights for optimizing comprehensive perinatal management.

## 2 Materials and methods

### 2.1 Study design

**Figure 1:**
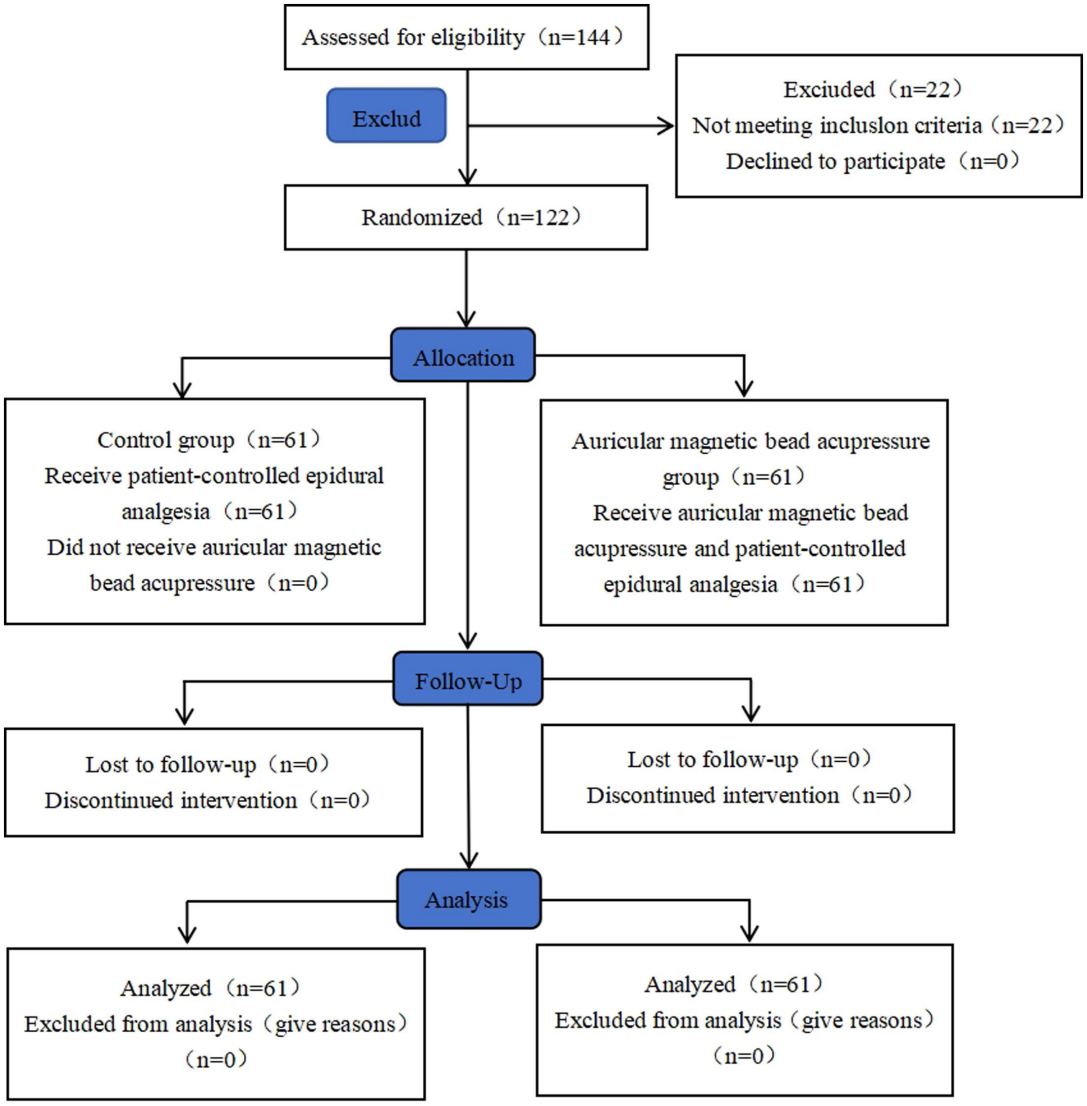
Consort flow diagram of the trial design.

This study was a prospective, randomized controlled clinical trial aimed at assessing the efficacy of AMBA in enhancing postpartum sleep quality and reducing fatigue among primiparous women. Additionally, the trial sought to observe the preventive effects of AMBA on epidural-related maternal fever (ERMF) and to explore potential underlying mechanisms. The research was conducted at the First Affiliated Hospital of Guizhou University of Traditional Chinese Medicine from January 2025 to December 2025. The study protocol received approval from the Ethics Committee of the First Affiliated Hospital of Guizhou University of Traditional Chinese Medicine (Approval No.: KS2024174) and was registered with the International Traditional Medicine Clinical Trial Registry (ITMCTR; No.: ITMCTR2025002105). Written informed consent was obtained from all participants. A comprehensive study flowchart is presented in (Figure 1).

### 2.2 Participants

A total of 144 eligible primiparous women were recruited for the study. The inclusion criteria were: age 18–35 years; gestational age 37–42 weeks; singleton pregnancy with cephalic presentation; American Society of Anesthesiologists (ASA) physical status I–II; and no contraindications to epidural analgesia or vaginal delivery. The exclusion criteria were: prenatal temperature ≥37.5 °C; severe obstetric complications; use of antibiotics or non-steroidal anti-inflammatory drugs before labor; hepatic or renal insufficiency; major neurological or psychiatric disorders; known, pre-existing sleep disorders (e.g., moderate-to-severe sleep apnea or restless legs syndrome) or regular use of sedative-hypnotics/psychotropic medications within 1 week before labor; known allergy or contraindication to the epidural analgesic regimen or study materials; substance abuse; inability to complete questionnaires; failed epidural analgesia; or conversion to cesarean section.

### 2.3 Randomization and blinding

The sample size was calculated based on the primary outcome—the Athens Insomnia Scale (AIS) score on postpartum day 5. According to a literature review and preliminary clinical observations, the estimated mean AIS score in the control group (epidural analgesia alone) was assumed to be 6.5 ± 3.1. We hypothesized that AMBA intervention would reduce the AIS score by approximately 1.5 points (clinically meaningful difference). With a two-sided significance level (*α*) of 0.05 and a statistical power (1–*β*) of 0.8, the sample size was calculated using the formula for comparing two independent means. The initial calculation yielded approximately 61 participants per group. Anticipating a potential dropout rate of ~15% (due to factors such as conversion to cesarean section, epidural failure, or withdrawal), the adjusted sample size per group was determined as 72. Thus, we planned to enroll 72 participants per group, totaling 144 subjects, which also ensures adequate power for the analysis of key secondary outcomes (ERMF incidence, fatigue scores). This sample size also meets the requirement for preliminary analysis of the primary outcome. Participants were randomly assigned to either the intervention group (AMBA + Patient-Controlled Epidural Analgesia, PCEA) or the control group (PCEA). The randomization sequence was generated by research department personnel using SPSS Statistics (version 25.0) with block randomization to ensure balanced group sizes. Allocation concealment was strictly implemented using sequentially numbered, opaque, sealed envelopes (SNOSE). Due to the nature of the non-pharmacological intervention, blinding of participants and the practitioners performing the acupressure was not feasible (i.e., open-label design). However, to minimize bias, outcome assessors (responsible for collecting biological samples and questionnaires) and data analysts were kept blinded to group allocation throughout the study.

### 2.4 Intervention

**Figure 2:**
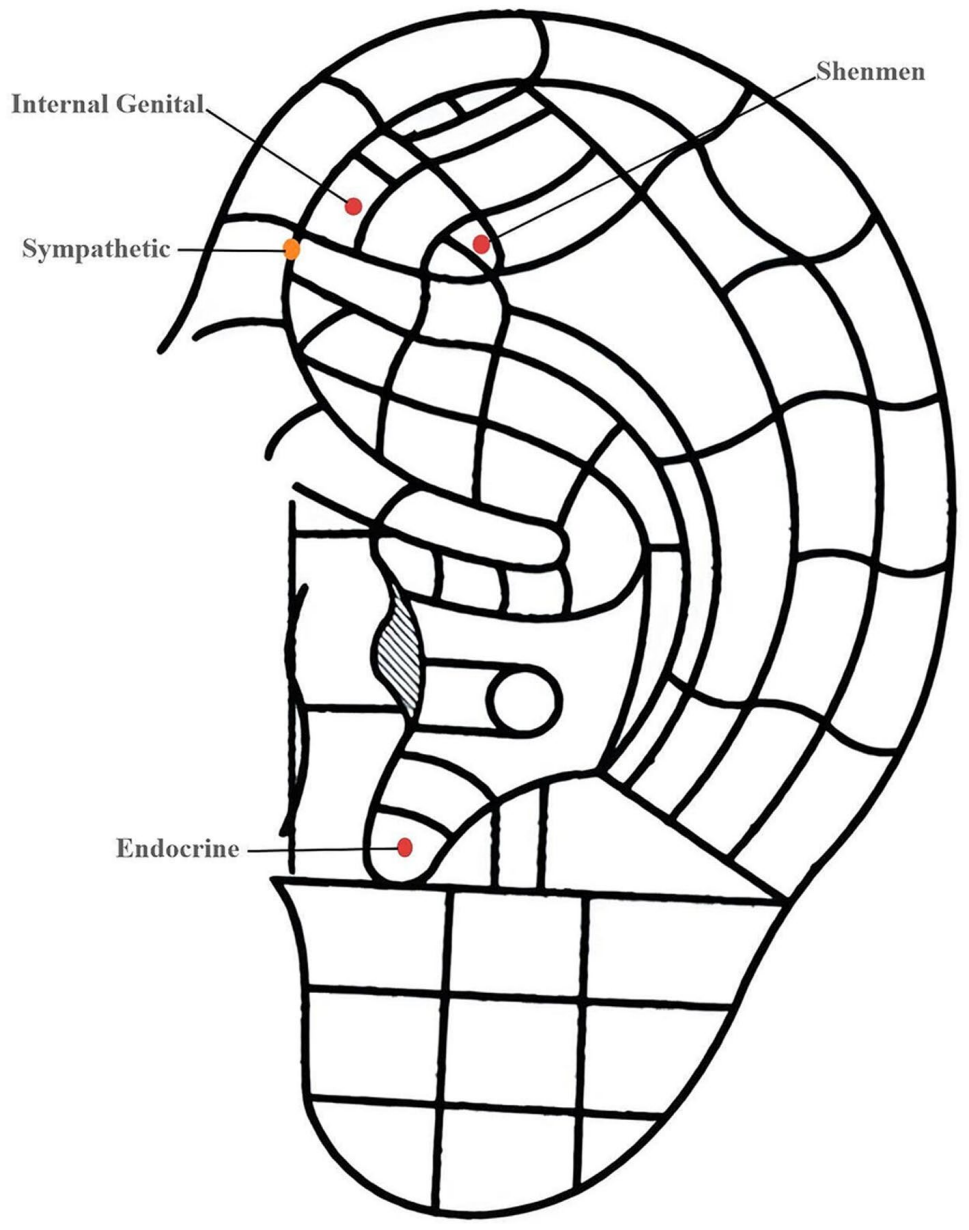
Schematic diagram of auricular magnetic bead acupressure (AMBA) intervention.

Upon entering the delivery room, an intravenous line was established, and a compound sodium lactate solution, maintained at 37 °C, was administered at a rate of 8 mL/kg/h. The ambient temperature in both the labor and delivery rooms was controlled within the range of 25–28 °C. Labor analgesia was initiated for both groups when cervical dilation reached 1–2 cm, with epidural catheterization performed by a single anesthesiologist. The patient-controlled epidural analgesia (PCEA) regimen included 0.075% ropivacaine combined with sufentanil at a concentration of 0.5 μg/mL (Yichang Humanwell Pharmaceutical, Yichang, Hubei, China). This regimen involved a 5 mL loading dose, a continuous background infusion at 8 mL/h, a patient-controlled bolus of 3 mL, and a lockout interval of 30 min. Analgesia was sustained until the completion of delivery. Participants in the control group received PCEA and routine intrapartum care/monitoring only, without any auricular bead placement or acupressure manipulation. The AMBA group received PCEA in conjunction with auricular magnetic bead acupressure, administered by a traditional Chinese medicine (TCM) physician with 20 years of experience. Magnetic beads were applied to specific auricular points, namely Shenmen, Internal Genitalia, Endocrine, and Sympathetic, with an additional bead placed on the corresponding area on the back of the ear (Suzhou Medical Supplies Factory, Hwato Magnetic Beads for Acupressure, Suzhou, Jiangsu, China). Following application, the beads were pressed and rotated using the thumb and index finger until the parturient reported sensations of soreness, numbness, or slight pain. Pressure was applied at 20-min intervals, with each application lasting for one minute (Figure 2). The anesthesiologist evaluated analgesic efficacy on an hourly basis using the Visual Analogue Scale (VAS) for pain, where a score of 0 indicated “no pain” and a score of 10 indicated “the worst pain imaginable.” This approach ensured adequate analgesia and facilitated timely adjustments to the analgesic regimen, with participants not meeting the standard being excluded from the study.

### 2.5 Outcome measures

#### 2.5.1 Primary outcomes

##### 2.5.1.1 Subjective assessment of sleep quality and fatigue

Sleep quality was assessed using two validated scales. The Pittsburgh Sleep Quality Index (PSQI) was administered upon admission (prenatal baseline) to evaluate general sleep quality over the preceding month. The PSQI global score ranges from 0 to 21, with higher scores indicating poorer sleep quality. The scoring criteria were as follows: a score of 0–5 indicates “very good” sleep quality, 6–10 indicates “fairly good” sleep quality, 11–15 indicates “general” sleep quality, and 16–21 indicates “very poor” sleep quality. Because the PSQI evaluates sleep over the preceding month, it is well suited for baseline characterization but may be less sensitive to short-term changes immediately after delivery.

Postpartum sleep disturbances were assessed on postnatal day 2 (PND 2) and postnatal day 5 (PND 5) using the Athens Insomnia Scale (AIS). The AIS comprises eight items, each rated on a scale from 0 to 3, resulting in a total score ranging from 0 to 24. According to established cut-off values, a total score of less than 4 suggests the absence of a sleep disorder, a score between 4 and 6 indicates suspected insomnia, and a score greater than 6 confirms the presence of insomnia. The AIS focuses on recent insomnia symptoms and is more sensitive to short-term postpartum changes; using PSQI repeatedly within a short postpartum interval would also introduce substantial overlap in its 1-month recall window.

Fatigue severity was evaluated on PND 2 and PND 5 using the Postpartum Fatigue Scale (PFS), a 10-item self-report measure developed by Milligan et al. (13). The Chinese version, translated by Qian et al. (14), was utilized, demonstrating good reliability (Cronbach’s *α* = 0.818) (15). The scale encompasses two dimensions: physical and mental fatigue. Each item is rated on a 4-point Likert scale ranging from 1 (“not at all”) to 4 (“always”), reflecting fatigue levels over the preceding week. The total score ranges from 10 to 40, with higher scores indicating greater fatigue severity. Fatigue severity was categorized as follows: a score of 10 indicates no fatigue, 11–14 denotes mild fatigue, 15–20 represents moderate fatigue, and 21–40 signifies severe fatigue.

#### 2.5.2 Secondary outcomes

##### 2.5.2.1 Inflammatory cytokines and sleep-related molecules

To evaluate inflammatory levels and neuroendocrine function, venous blood samples (2–3 mL) were obtained from parturients at the following time points: T0 (upon admission/before analgesia), T1 (immediately post-fetal delivery), T2 (postnatal day 2), and T3 (postnatal day 5). Blood samples were collected into vacuum tubes without anticoagulant. Sample processing involved centrifuging all blood samples at 3,000 × g for 15 min at 4 °C within 30 min of collection to isolate serum. The serum was then aliquoted into sterile cryotubes and promptly stored at −80 °C in an ultra-low temperature freezer for long-term preservation until batch analysis, thereby preventing repeated freeze–thaw cycles. Assay methods included quantification of serum concentrations of inflammatory cytokines (IL-6, IL-1β) and molecules related to sleep–wake regulation (serotonin, melatonin, cAMP, GABA) using enzyme-linked immunosorbent assay (ELISA, Shanghai Jianglai Biotechnology, Shanghai, China). All procedures were meticulously conducted in accordance with the manufacturer’s instructions. To minimize inter-assay variability, all samples from the same participant across different time points were analyzed on the same assay plate. Laboratory personnel responsible for conducting the assays and analyzing the data were blinded to the group allocation of the samples.

##### 2.5.2.2 Incidence of epidural-related maternal fever (ERMF)

Tympanic temperature was assessed utilizing a consistent model of calibrated infrared tympanic thermometer, specifically the Braun ThermoScan 7. These measurements were conducted by delivery room nurses who were trained to ensure the use of a new probe cover for each measurement and to manipulate the pinna by pulling it upward and backward to straighten the ear canal. Temperature readings were recorded at the following intervals: prior to the administration of epidural analgesia (baseline, T0), 2 h post-initiation of analgesia (T1), 4 h post-initiation of analgesia (T2), and 1 h following fetal delivery (T3). The diagnosis of Epidural-Related Maternal Fever (ERMF) was established if the tympanic temperature reached or exceeded 37.5 °C at any consecutive measurement points (T1, T2, T3) and persisted for more than 30 min. The incidence and percentage of parturients who developed ERMF were documented for each cohort.

##### 2.5.2.3 Pain VAS scores at different time points and analgesia record

Pain intensity was evaluated utilizing the Visual Analogue Scale (VAS) at specific time points: prior to the administration of analgesia (Ta), at 3 cm of cervical dilation (Tb), at complete cervical dilation (10 cm, Tc), and following fetal delivery (Td). Concurrently, data were collected directly from the patient-controlled epidural analgesia (PCEA) pump, including the number of effective PCEA demands, which is defined as the count of successful bolus deliveries within the lockout interval, and the total analgesic drug consumption, calculated as the total volume in milliliters of the 0.075% ropivacaine and sufentanil solution utilized. The duration of analgesia was defined as the time interval, measured in minutes, from the successful administration of the initial PCEA loading dose to the moment of fetal delivery.

##### 2.5.2.4 Placental pathological examination

Placental tissues were collected by midwives immediately after delivery and transported to the pathology department within 1 h (maintained at 4 °C). Tissue samples were obtained from the maternal and fetal surfaces, membranes, and umbilical cord following a standardized protocol. Specimens were processed routinely, sectioned, and stained with hematoxylin and eosin (H&E). Two experienced pathologists, blinded to group allocation, independently evaluated all slides. Histologic chorioamnionitis (HCA) was diagnosed according to established criteria as neutrophil infiltration in any of the following tissues: chorion, amnion, decidua, or umbilical cord. The presence or absence of HCA was recorded for each participant.

##### 2.5.2.5 Labor Progress and obstetric interventions

Data on labor progress and interventions were systematically documented by delivery room nurses or midwives who received uniform training. The duration of labor was categorized into two phases: the active phase, defined as the period from cervical dilation of 6 cm to full dilation (10 cm), and the second stage, defined as the period from full dilation to the delivery of the fetus. Time intervals were precisely recorded to the nearest minute using standardized electronic timers available in the delivery room. Regarding oxytocin administration, the total dose, measured in Units (U), was recorded from the onset of labor to the completion of delivery for the purposes of labor augmentation or induction. Oxytocin was diluted in 0.9% saline and administered intravenously through an infusion pump, beginning at an initial rate of 2 milliunits per minute (mU/min). The dosage was adjusted every 15 to 30 min based on uterine contraction patterns until effective contractions were achieved, defined as 3 to 5 contractions per 10 min, each lasting 40 to 60 s, with a maximum allowable dose of 20 mU/min. The total administered dose was calculated by multiplying the infusion rate (mU/min) by the infusion time (min) and converting the result into Units (U). Number of vaginal examinations: The total number of vaginal examinations performed from admission to fetal delivery to assess cervical dilation and fetal descent was recorded. All data were recorded in real-time on standardized Case Report Forms (CRFs).

### 2.6 Statistical analysis

Data were analyzed using SPSS statistical software (version 25.0). Normally distributed continuous data are presented as mean ± standard deviation (x ± s) and were compared between groups using the independent samples *t*-test. Non-normally distributed continuous data are presented as median (interquartile range) and were compared using the Mann–Whitney U test. Categorical data are presented as number (percentage) and were compared using the Chi-square test or Fisher’s exact test, as appropriate. Intragroup comparisons for repeated measures data (e.g., temperature, VAS scores, cytokine levels) were performed using the paired *t*-test or Wilcoxon signed-rank test. A two-sided *p*-value < 0.05 was considered statistically significant.

## 3 Results

### 3.1 Patient characteristics and baseline information

Initially, 144 primiparous women were enrolled. After exclusions (Figure 1), 122 women (61 per group) completed the study and were included in the final analysis. Baseline characteristics are presented in Table 1 and were comparable between groups (*p* > 0.05), including age, gestational age, anthropometrics, baseline SpO₂, baseline pulse, and educational level.

**Table 1:** Baseline characteristics of the two groups of primiparous women.

Variable	Control (*n* = 61)	AMBA (*n* = 61)	*p*-value
Age (years)	26.44 ± 3.04	26.75 ± 3.36	0.592
Educational level			
High school or below	16	17	0.839
College or above	45	44	
Gestational age (weeks)	38.82 ± 1.19	38.62 ± 1.27	0.379
Height (cm)	159.00 (157.00,162.00)	160.00 (156.00,163.00)	0.977
Weight (kg)	59.80 ± 9.64	64.00 ± 11.85	0.064
BMI (kg/m^2^)	23.24 (20.01, 25.00)	24.03 (21.08, 27.06)	0.055
Baseline SpO₂(%)	99.00 (99.00, 99.00)	99.00 (99.00, 99.00)	0.816
Baseline Pulse (beats/min)	85.90 ± 13.32	85.49 ± 10.62	0.851

Values are presented as mean ± standard deviation, median (interquartile range), or number (percentage). *p*-values were derived from independent *t*-test (for normally distributed data), Mann–Whitney U test (for non-normally distributed data), or Chi-square test (for categorical data), as appropriate.

### 3.2 Expression levels of sleep-related biomarkers

#### 3.2.1 Serum 5-HT and melatonin levels

The comparative analysis of serum serotonin (5-HT) and melatonin (MT) levels between the two groups is detailed in Tables 2, 3, respectively. At baseline, there were no statistically significant differences in 5-HT or MT levels between the groups (*p* > 0.05). Postpartum, significant changes in serum 5-HT levels were observed in both groups. Specifically, 5-HT levels significantly decreased on postnatal day 2 (PND2) compared to baseline in both the control and AMBA groups (*p* < 0.05, Table 2). However, intergroup comparisons indicated that the AMBA group had significantly higher 5-HT levels than the control group at both PND2 (*p* = 0.0006) and PND5 (*p* = 0.0202). Regarding serum melatonin, a significant reduction from baseline was observed at PND2 in the control group (*p* < 0.05), but not in the AMBA group (Table 3). Intergroup comparisons demonstrated that MT levels in the AMBA group were significantly higher than those in the control group at PND2 (*p* = 0.0341) and PND5 (*p* = 0.0086).

**Table 2:** Comparison of serum 5-HT levels at different time points between the two groups.

Group	*N*	5-HT (ng/mL)		
		Baseline	PND2	PND5
Control	61	165.50±38.55	125.20±23.85**	142.50±28.53
AMBA	61	166.70±46.95	155.26 ± 33.39**	164.78 ± 36.68
*p*-value		0.9212	**0.0006**	**0.0202**

Data are presented as mean ± standard deviation. 5-HT, 5-hydroxytryptamine (serotonin); PND, postnatal day. *p*-value for between-group comparisons (Control vs. AMBA). **p < 0.05 for within-group comparisons vs. baseline.Bold values indicate statistically significant between-group differences (*p* < 0.05).

**Table 3:** Comparison of Serum MT Levels at different time points between the two groups.

Group	*N*	MT (pg/mL)		
		Baseline	PND2	PND5
Control	61	73.89 ± 12.79	66.84 ± 10.08**	69.27 ± 5.80
AMBA	61	79.65±12.05	73.38±10.18	76.94±10.33
*p*-value		0.1229	**0.0341**	**0.0086**

Data are presented as mean ± standard deviation. MT, melatonin; PND, postnatal day. *p*-value for between-group comparisons (Control vs. AMBA). **p < 0.05 for within-group comparisons vs. baseline.Bold values indicate statistically significant between-group differences (*p* < 0.05).

#### 3.2.2 Serum cAMP and GABA levels

**Figure 3:**
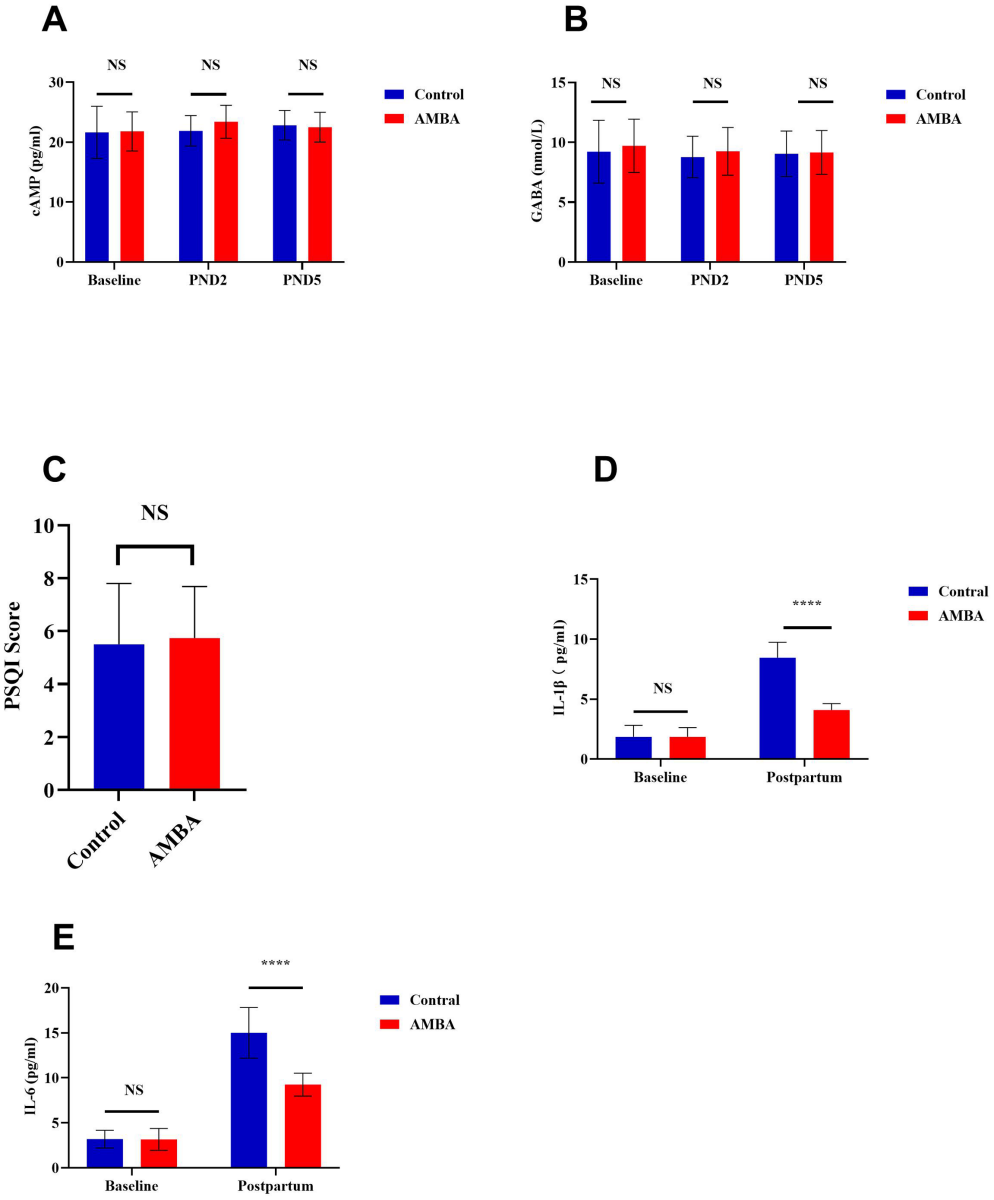
Effects of auricular magnetic bead acupressure (AMBA) on serum biomarkers, sleep quality, and inflammatory cytokine levels in parturients.

As illustrated in Figures 3A,B, no significant differences were observed in serum cyclic adenosine monophosphate (cAMP) or gamma-aminobutyric acid (GABA) levels between the two groups at any of the assessed time point (baseline, PND2, PND5; all *p* > 0.05).

### 3.3 Subjective assessments of sleep and fatigue

The baseline sleep quality, evaluated using the total Pittsburgh Sleep Quality Index score, also revealed no significant differences between the groups (Figures 3C, *p* > 0.05). A summary of postpartum subjective sleep and fatigue assessments is provided in (Table 4). On postpartum day 2 (PND 2), no statistically significant differences were found between the control and AMBA groups in either the Athens Insomnia Scale score or the PFS total score (*p* > 0.05). However, by PND 5, the AMBA group demonstrated a significantly lower Athens Insomnia Scale score compared to the control group (*p* = 0.006). Additionally, the PFS total score was significantly reduced in the AMBA group relative to the control group (*p* < 0.001). Concerning the distribution of fatigue severity on PND 5, the proportion of mothers experiencing “Severe Fatigue” was significantly lower in the AMBA group compared to the control group (8.2% vs. 31.1%, *p* = 0.0004). In contrast, the proportion categorized as having “Mild Fatigue” was significantly higher in the AMBA group (34.4% vs. 18.0%, *p* = 0.0002).

**Table 4:** Postpartum subjective sleep and fatigue assessments.

Assessment (Scale)	Timepoint	Control (*n* = 61)	AMBA (*n* = 61)	*p*-value
Athens Insomnia Scale (AIS) Score	PND 2	5.77 ± 3.81	5.26 ± 3.62	0.405
	PND 5	6.03 ± 3.13	4.72 ± 2.77	0.006
Postpartum Fatigue Scale (PFS) total score	PND 2	22.56 ± 8.59	19.93 ± 7.17	0.503
	PND 5	20.40 ± 0.89	16.13 ± 2.91	<0.001
Fatigue severity distribution, *n* (%)				
Mild Fatigue	PND 5	11 (18.0)	21 (34.4)	0.0002
Moderate Fatigue	PND 5	31 (50.8)	35 (57.4)	0.6030
Severe Fatigue	PND 5	19 (31.1)	5 (8.2)	0.0004

Data are presented as mean ± standard deviation. *p*-value for between-group comparisons (Control vs. AMBA).

### 3.4 Expression levels of serum inflammatory factors

The concentrations of serum inflammatory cytokines are presented in (Figures 3D,E). Initially, no significant differences were detected in serum IL-1β or IL-6 levels between the two groups. By postnatal day 5, the concentrations of both IL-1β and IL-6 had significantly increased from baseline levels across all participants. Notably, the AMBA group demonstrated significantly lower concentrations of both IL-1β and IL-6 compared to the control group on postnatal day 5 (*p* < 0.0001).

### 3.5 Temperature changes at each time point and incidence of ERMF in Parturients

The alterations in tympanic membrane temperature and the incidence of ERMF are detailed in Table 5. At baseline (T0; *p* = 0.128), there was no significant between-group difference. At 2 h post-analgesia (T1; *p* = 0.039), tympanic temperature was marginally higher in the control group than in the AMBA group, although the absolute difference was small. At 4 h post-analgesia (T2; *p* < 0.001) and 1 h post-delivery (T3; *p* = 0.004), the control group demonstrated significantly higher tympanic temperatures compared with the AMBA group. Within the control group, temperatures at T2 and T3 were significantly elevated relative to T0 (both *p* < 0.01), whereas the AMBA group showed no significant temperature changes from baseline at any assessed time point. Additionally, the incidence of ERMF was significantly lower in the AMBA group than in the control group (8.2% vs. 24.6%; *p* = 0.027).

**Table 5:** Comparison of tympanic membrane temperature and incidence of ERMF between the two groups.

Group	*N*	T0 (°C)	T1 (°C)	T2 (°C)	T3 (°C)	ERMF (*n*, %)
Control	61	36.59 ± 0.25	36.41 ± 0.17	37.21 ± 0.38**	37.23 ± 0.31**	15 (24.59%)
AMBA	61	36.52 ± 0.24	36.37 ± 0.25	36.93 ± 0.36	37.06 ± 0.35	5 (8.19%)
*p*-value		0.1283	0.0389	**<0.0001**	**0.0044**	**0.0265**

Data are presented as mean ± standard deviation or number (percentage). T0, before analgesia; T1, 2 h after analgesia; T2, 4 h after analgesia; T3, 1 h after fetal delivery; ERMF, epidural-related maternal fever. *p*-values in the bottom row are for between-group comparisons (Control vs. AMBA) at each time point, derived from independent *t*-test (for temperature) or Chi-square test (for ERMF incidence). *p* < 0.01 for within-group comparisons vs. T0 (paired *t*-test).**Indicates *p* < 0.01 for within-group comparisons versus T0 (baseline). Bold values indicate statistically significant between-group differences (*p* < 0.05).

### 3.6 Placental histopathological examination

**Figure 4:**
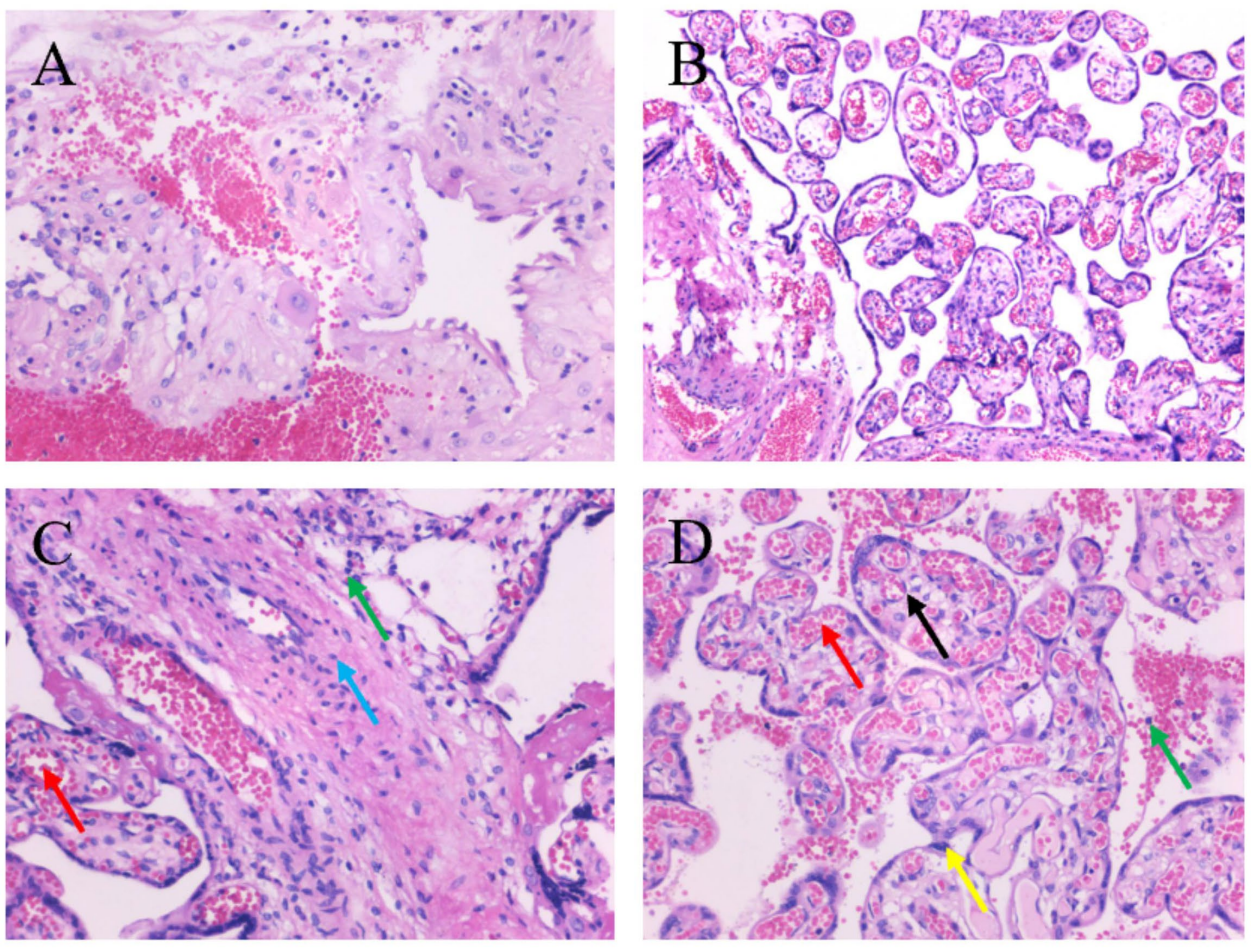
Histopathological examination of placental tissues.

To evaluate histological chorioamnionitis (HCA), characterized mainly by neutrophilic infiltration into the chorion, amnion, decidua, or umbilical cord, histopathological analysis of placental tissues was conducted for both groups. Figure 4 shows representative photomicrographs of placental sections. Examination at 100 × and 200 × magnification showed no neutrophilic infiltration in the placental tissues from either group. Additionally, the control group had chorionic villi with congested capillaries, narrowed lumina, mild perivillous fibrin deposition, and scattered lymphocytic infiltration (Figures 4A,C). Conversely, the AMBA group showed enlarged and irregularly shaped villi, a loosely arranged intervillous space, focal discontinuity of the syncytiotrophoblast layer, dilated and congested villous capillaries, and scant lymphocytic infiltration (Figures 4B,D). Therefore, no significant difference between the groups was found in the diagnosis of HCA.

### 3.7 Analgesic efficacy and drug consumption

Analgesic outcomes and drug consumption are summarized in Table 6. At Ta (before analgesia), VAS scores differed between groups (*p* = 0.006), whereas no significant differences were observed at Tb or Tc (*p* > 0.05). At Td (after fetal delivery), the AMBA group had significantly lower VAS scores than the control group (*p* < 0.001). The number of effective PCEA demands did not differ significantly between groups (*p* = 0.714). Total analgesic consumption (total volume of standardized PCEA solution) was significantly lower in the AMBA group than in the control group (57.8 ± 8.6 mL vs. 73.0 ± 12.5 mL; *p* < 0.001).

**Table 6:** Comparison of analgesic conditions and drug consumption between the two groups.

Variable	Timepoint	Control (*n* = 61)	AMBA (*n* = 61)	*p*-value
VAS score	T_a_	7.0 (5.0, 7.0)	7.0 (6.0, 8.0)	0.006
	T_b_	4.0 (3.0, 4.0)	3.0 (3.0, 4.0)	0.130
	T_c_	6.0 (6.0, 7.0)	6.0 (6.0, 7.0)	0.073
	T_d_	6.0 (5.0, 6.0)	5.0 (5.0, 6.0)	<0.001
PCEA requests (times)	Total (T_d_)	4.0 (3.5, 5.0)	4.0 (4.0, 5.0)	0.714
Analgesic dosage (mL)	Total (T_d_)	73.0 ± 12.50	57.8 ± 8.60	<0.001

VAS, Visual Analogue Scale; PCEA, Patient-Controlled Epidural Analgesia. Data are presented as median (25th percentile, 75th percentile) for VAS scores and PCEA requests, and as mean ± standard deviation for analgesic dosage. Ta, Tb, Tc, and Td are defined as the pain assessment timepoints: Ta (before analgesia); Tb (at 3 cm cervical dilation); Tc (at full cervical dilation, 10 cm); Td (after fetal delivery).

### 3.8 Labor progress and obstetric interventions

The progress of labor and associated interventions are compared in (Table 7). The duration of the active phase of labor was comparable between the control and AMBA groups (208.7 ± 31.13 min vs. 202.9 ± 26.52 min; *p* = 0.6525). In contrast, the duration of the second stage of labor was significantly shorter in the AMBA group compared to the control group (46.50 ± 7.43 min vs. 58.05 ± 9.39 min; *p* < 0.0001). Regarding obstetric interventions, the AMBA group required a significantly lower total dose of oxytocin (median: 0.50 U vs. 1.40 U; *p* < 0.0001) and underwent significantly fewer vaginal examinations (median: 5.00 vs. 7.00; *p* < 0.0001).

**Table 7:** Comparison of labor progress and interventions between two groups of primiparous women.

Group	*N*	Active phase (Min)	Second stage (Min)	Oxytocin dose (U)	Vaginal exams (*N*)
Control	61	208.7 ± 31.13	58.05 ± 9.39	1.40 (1.20, 1.70)	7.00 (6.50, 8.00)
AMBA	61	202.9 ± 26.52	46.50 ± 7.43	0.50 (0.10, 0.75)	5.00 (4.50, 6.00)
*p*		0.6525	**<0.0001**	**<0.0001**	**<0.0001**

Values are presented as mean ± standard deviation for normally distributed data (Active Phase, Second Stage) or median (interquartile range) for non-normally distributed data (Oxytocin Dose, Vaginal Exams). *p*-values are for between-group comparisons (Control vs. AMBA), derived from independent *t*-test (for Active Phase and Second Stage) or Mann–Whitney U test (for Oxytocin Dose and Vaginal Exams)Bold values indicate statistically significant between-group differences (*p* < 0.05)..

## 4 Discussion

This randomized controlled trial suggests that auricular magnetic bead acupressure (AMBA) may improve early postpartum sleep and fatigue in primiparous women receiving epidural labor analgesia, and may reduce the incidence of epidural-related maternal fever (ERMF). Postpartum sleep disturbances and severe fatigue are prevalent issues that impair early maternal recovery and quality of life, often creating a vicious cycle with pain and stress (16, 17). Traditionally, the management of parturients receiving epidural analgesia has concentrated more on physiological complications, such as ERMF, with comparatively less attention given to their postpartum sleep experience (18). Our findings indicate that AMBA is associated with improved sleep and reduced fatigue during the early recovery period, which may be clinically meaningful given links between early postpartum sleep disruption and adverse maternal outcomes (19, 20).

Regarding fatigue, the between-group differences in PFS scores should be interpreted in terms of clinical relevance. Because a universally accepted minimal clinically important difference (MCID) for the Postpartum Fatigue Scale has not been firmly established across postpartum populations and timeframes, we additionally describe the magnitude of change using fatigue severity categories (mild/moderate/severe) and effect-size considerations to aid clinical interpretation.

In order to investigate the potential biological mechanisms underlying sleep enhancement facilitated by AMBA, we conducted a detailed analysis of serum biomarkers intimately associated with sleep–wake regulation. Our findings indicate that the AMBA group exhibited significantly elevated levels of melatonin and serotonin postpartum. Melatonin, an endogenous sleep inducer, plays a critical role in maintaining normal sleep architecture (21, 22), while serotonin (5-HT) is a pivotal neurotransmitter involved in the regulation of mood, sleep initiation, and pain perception (23). The pain and stress associated with labor, along with the inflammatory response potentially induced by epidural analgesia, can disrupt neuroendocrine homeostasis (24, 25). The observed attenuation in the rise of serum IL-6 and IL-1β levels within the AMBA group suggests a systemic anti-inflammatory effect. Consequently, we hypothesize that AMBA may mitigate the disruption of the neuro-immune-endocrine network through its dual actions of anti-inflammation and autonomic nervous system regulation, thereby preserving the normal functioning of the melatonin and serotonin systems. This mechanism may be pivotal in breaking the vicious cycle of “pain-stress-inflammation-sleep disturbance” (26). However, our biomarker findings are associative and do not provide direct evidence that these biological changes mediate the observed clinical outcomes; mechanistic studies are needed to establish causality.

It is noteworthy that the improvement in sleep with AMBA was accompanied by a significant reduction in ERMF incidence and total analgesic consumption. This suggests that the sleep benefits likely result from the synergy of multiple factors: the reduction of ERMF directly removes fever as a physiological disruptor of sleep; the sparing effect on analgesics, particularly the relative reduction in opioids, might minimize potential drug-induced interference with sleep architecture (27); furthermore, the additional analgesic effect provided by AMBA after fetal delivery directly alleviates immediate postpartum discomfort, creating more favorable conditions for sleep (28, 29).

An interesting finding is that despite significant systemic anti-inflammatory effects, histopathological examination of placental tissues revealed no significant difference in inflammatory infiltration between the groups. This finding instead supports the notion that the site of AMBA’s action may lie more within the central nervous system and systemic regulatory pathways rather than specific peripheral tissues. This aligns with the modern medical interpretation that auricular therapy influences the nucleus tractus solitarius in the brainstem via stimulation of the auricular branch of the vagus nerve, thereby broadly modulating autonomic nervous function, highlighting its unique value in regulating systemic psychosomatic states (30).

Strengths of this study include the randomized design with allocation concealment, a standardized epidural analgesia protocol applied to both groups, and the combination of validated patient-reported outcomes with objective biomarkers and placental histopathology. Importantly, AMBA represents a feasible integrative, non-pharmacological adjunct that can be implemented within routine obstetric anesthesia care, supporting a multimodal approach to postpartum symptom management.

### 4.1 Limitations of this study

This randomized, allocation-concealed trial applied a standardized epidural protocol and combined validated patient-reported outcomes with objective biomarkers/placental histopathology. AMBA is a feasible non-pharmacological adjunct that can be integrated into routine obstetric anesthesia care to support multimodal postpartum symptom management. Nevertheless, several limitations should be noted. First, the single-center design in a traditional Chinese medicine hospital and the moderate sample size may limit generalizability to other clinical settings. Second, participants and practitioners could not be blinded and no sham procedure was used, so expectancy/placebo effects cannot be excluded. Third, some participants were excluded after randomization, which may introduce attrition bias. Fourth, sleep outcomes relied primarily on subjective scales; future studies should incorporate objective measures such as actigraphy or polysomnography. Fifth, tympanic temperature measurement may introduce variability despite standardized procedures. Finally, repeated-measures outcomes were analyzed using pairwise comparisons; mixed-effects modeling could better account for within-subject correlation and baseline differences. The specific neural circuits and molecular mechanisms through which AMBA exerts its effects also require further elucidation in dedicated mechanistic studies.

## 5 Conclusion

In conclusion, this randomized trial suggests that adjunctive auricular magnetic bead acupressure (AMBA) may improve early postpartum sleep quality and fatigue and may reduce the incidence of epidural-related maternal fever in primiparous women receiving epidural labor analgesia. The observed biomarker changes (e.g., melatonin, serotonin, and inflammatory markers) are consistent with an association between AMBA and neuroendocrine/immune modulation; however, these findings do not establish a causal mechanism. As a non-invasive and easily applicable adjunct, AMBA may be a promising option for integrative perinatal care. Future multicenter studies with larger sample sizes, sham controls, and objective sleep monitoring are warranted to confirm efficacy and clarify mechanisms.

## Data Availability

The raw data supporting the conclusions of this article will be made available by the authors, without undue reservation.
